# Prediction of Signal Peptides in Proteins from Malaria Parasites

**DOI:** 10.3390/ijms19123709

**Published:** 2018-11-22

**Authors:** Michał Burdukiewicz, Piotr Sobczyk, Jarosław Chilimoniuk, Przemysław Gagat, Paweł Mackiewicz

**Affiliations:** 1 Faculty of Mathematics and Information Science, Warsaw University of Technology, 00-661 Warszawa, Poland; michalburdukiewicz@gmail.com; 2 Department of Mathematics, Wrocław University of Technology, 50-370 Wrocław, Poland; Piotr.Sobczyk@pwr.edu.pl; 3 Department of Genomics, University of Wrocław, 50-383 Wrocław, Poland; jaroslaw.chilimoniuk@gmail.com (J.C.); przemyslaw.gagat@uwr.edu.pl (P.G.)

**Keywords:** apicomplexa, plasmodium, malaria, HSMM, hidden semi-Markov model, signal peptides

## Abstract

Signal peptides are N-terminal presequences responsible for targeting proteins to the endomembrane system, and subsequent subcellular or extracellular compartments, and consequently condition their proper function. The significance of signal peptides stimulates development of new computational methods for their detection. These methods employ learning systems trained on datasets comprising signal peptides from different types of proteins and taxonomic groups. As a result, the accuracy of predictions are high in the case of signal peptides that are well-represented in databases, but might be low in other, atypical cases. Such atypical signal peptides are present in proteins found in apicomplexan parasites, causative agents of malaria and toxoplasmosis. Apicomplexan proteins have a unique amino acid composition due to their AT-biased genomes. Therefore, we designed a new, more flexible and universal probabilistic model for recognition of atypical eukaryotic signal peptides. Our approach called signalHsmm includes knowledge about the structure of signal peptides and physicochemical properties of amino acids. It is able to recognize signal peptides from the malaria parasites and related species more accurately than popular programs. Moreover, it is still universal enough to provide prediction of other signal peptides on par with the best preforming predictors.

## 1. Introduction

### 1.1. Roles and Features of Signal Peptides

Eukaryotic proteins encoded in the nuclear genome are synthesized on free ribosomes in the cytosol or on ribosomes attached to the endoplasmic reticulum, and are subsequently transported to specific subcellular or extracellular compartments. The appropriate localization of a protein is essential for its proper function, and this information is contained in the protein as a short amino acid sequence called a targeting or sorting signal.

An N-terminal signal peptide (SP) is responsible for targeting proteins to the endomembrane system, including the endoplasmic reticulum and the Golgi apparatus, in which proteins undergo folding and posttranslational modifications such as glycosylation and phosphorylation [1]. The SP-bearing proteins can either stay inside these compartments, be inserted into cellular membranes or exported outside the cell. Proteins equipped with SPs play a crucial role in metabolism (β-galactosidase, pepsins) [2], maintenance of tissue structure (collagen) [3], immune response (interferons, interleukins) [4] and regulation of other organismal functions (prolactin, glucagon) [5].

**Figure 1:**
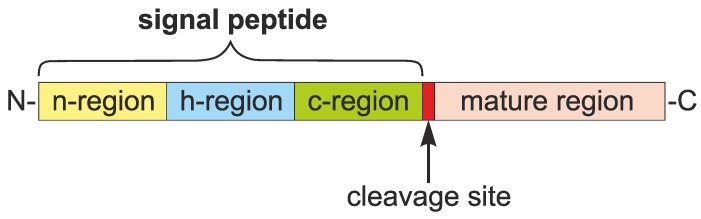
Organization of a typical signal peptide (SP). The lengths of SP regions are not drawn in scale.

Despite the low sequence similarity [6], a general structure of the SP was proposed and it includes three main parts: n-region, h-region and c-region (Figure 1) [7,8]. The SP starts with the n-region that is composed of a positively charged stretch of 5–8 amino acid residues. This part probably enforces the proper topology on a polypeptide during its translocation through the endoplasmic reticulum membrane based on the positive-inside rule [9]. The n-region is followed by the h-region, a stretch of 8–12 hydrophobic amino acids, which constitutes the core of the SP and usually forms an α-helix. The third component of the SP is the polar and uncharged c-region. This part is usually six residues long and ends with a cleavage site, at which a signal peptidase cuts the SP off during or after protein translocation into the lumen of the endoplasmic reticulum [10]. The cleavage site is characterized by a variable amino acid composition but typically contains small and neutral residues at −3 and −1 positions [11]. The cleavage site is, however, absent from some membrane proteins, in which the SP also acts as the first transmembrane domain. Such an uncleavable SP is sometimes referred as a signal anchor [12,13]. The amino acid composition and the length of these three characteristic regions vary between SPs and they influence the efficiency of protein translocation [14].

Understanding the structure of SPs and improving their prediction is of great importance in search of novel drugs and therapies. Equipping proteins with appropriate SPs or their modification can influence protein targeting to various subcellular or extracellular compartments, including substantial increase in their secretion [4,15]. SPs may also participate in tumour immunity, for example the presequence from midkine (a protein contributing to tumour progression) contains epitopes that are recognized by CD4+ T cells [16]. SPs can be potential medication targets in particular for malaria parasites belonging to the genus *Plasmodium* (family Plasmodiidae, phylum Apicomplexa) [17]. Their SPs differ in amino acid composition from typical SPs due to the strongly AT-biased genomes [18]. As a result, we can design therapies that target only these unique presequences and avoid interference with human host SPs. By acting on the malarian SPs, we can disturb many metabolic processes as the plasmodial SPs not only direct proteins into the endomembrane system and outside the cell but also into a digestive vacuole and a unique organelle characteristic only of Apicomplexa, i.e., apicoplast [19,20,21,22]. The apicoplast is a reduced four-membrane plastid that lost the photosynthetic function but still plays an important role in synthesis of lipids, heam and iron-sulphur clusters [20,21,23]. These Apicomplexa-specific compartments and their metabolic pathways are further potential targets for antimalarial drugs [24,25,26,27], which are intensively searched for due to increasing resistance of malaria parasites to the current drugs [28,29,30,31,32]. However, the existing software for SP prediction, trained and tested on proteins well-represented in available databases, does not perform satisfactorily in the case of Apicomplexa proteins.

### 1.2. Software Predicting Signal Peptides

**Figure 2:**
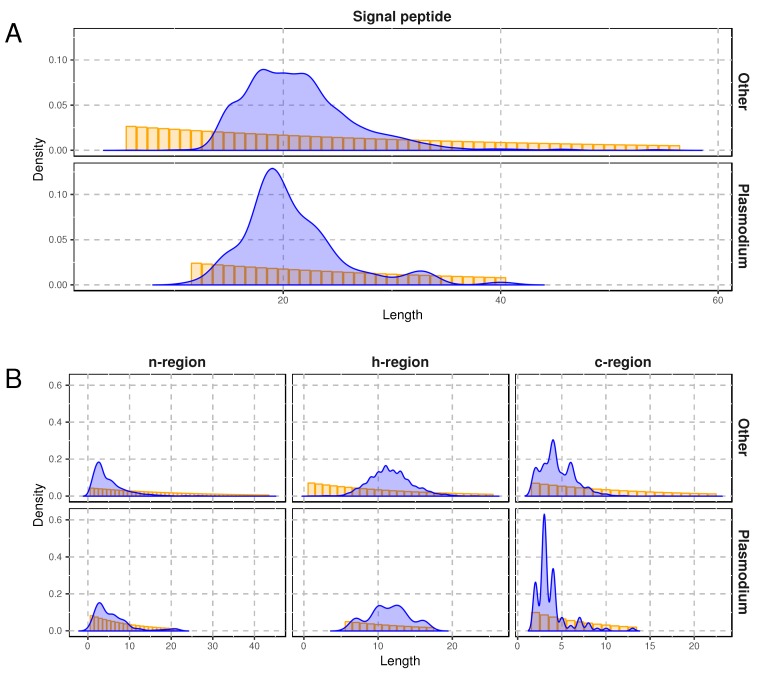
Distribution of lengths for SPs (**A**) and their regions (**B**) expressed in the number of amino acid residues for sequences with the representatives of the *Plasmodiidae* family (Plasmodium) and without them (Other). Yellow bars represent a fitted geometric distribution.

Since experimental methods for identification of targeting sequences are time-consuming and laborious, different computational approaches predicting targeting signals were developed. The software for SP prediction incorporates ’black-box’ models, such as: neural networks [33], support vector machines [34], Bayesian networks [35] or k-nearest neighbours [36], for which the decision rules are unknown to the user. More transparent algorithms are based on position matrices or their variants [34,37]. Others, e.g., Phobius, Philius and SignalP 3.0, use hidden Markov models (HMMs) [38,39,40] that try to reflect the structure of SP regions in their limited probabilistic frameworks. HMMs, however, imply a geometric distribution of the lengths of SP regions. Interestingly, we studied the distribution for the SP regions from the first work applying HMMs in SP prediction [41] and found that the length distribution for each region was not geometric (Figure 2).

The majority of SP predicting software uses the full amino acid alphabet of typical 20 residues. However, this approach treats all residues as separate entities and does not take into account the similarities between amino acids in terms of physicochemical properties. It may result in harmful oversimplification as the SP regions are in fact characterized by specific features of amino acid residues and not by the occurrence of particular amino acids. The only exception is BLOMAP [42], which uses a reduced alphabet of amino acids based on substitution matrices.

Moreover, the existing algorithms require a large number of sequences to be successfully learned. Although the learning sets are constructed to be as diverse as possible, the most frequent sequences dominate the creation of the decision rules. Consequently, the predictors usually accurately identify the most common SPs, but are ineffective in more atypical cases that are less represented in the training datasets. The commonly used rigid scheme of the SP organization (Figure 1) also does not characterize extremely long or short presequences, which constitute a substantial fraction of all SPs.

Therefore, we elaborated a new approach called signalHsmm. In order to enable the prediction of SPs with atypical amino acid composition, as in *Plasmodiidae*, we grouped amino acids based on their physicochemical properties. Instead of looking for patterns consisting of specific residues, we focused on more general features that are necessary for SPs to function properly. This approach was supported by the recent advancements in proteomics suggesting that the reduction of the amino acid alphabet could lead to better fold recognition [43,44]. Considering that one of the key features of SPs is α-helix, the use of a shorter amino acid alphabet may indeed improve the SP recognition.

In addition to the alphabet reduction, our algorithm is also based on hidden semi-Markov models. This flexible probabilistic framework does not assume the geometric distribution of the lengths of SP regions, but rather learns the distribution from the training dataset. Therefore, signalHsmm with hidden semi-Markov models and the reduced amino acid alphabet should be more general than its counterparts.

## 2. Results and Discussion

### 2.1. Performance of SignalHsmm Algorithm

**Figure 3:**
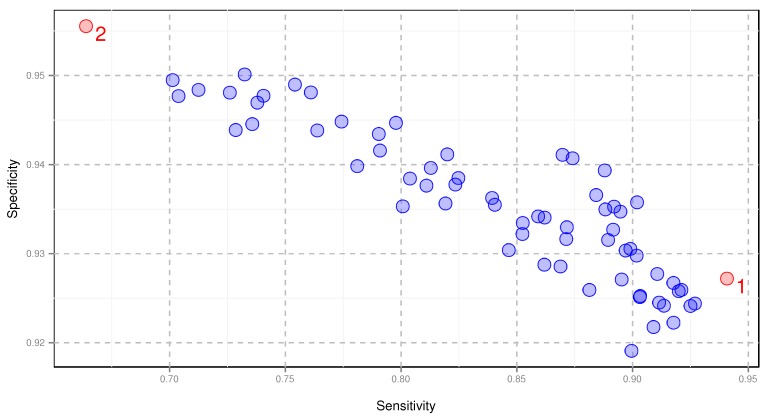
Sensitivity and specificity of amino acid encodings after cross-validation. (1) encoding providing the best sensitivity (AUC=0.9683, MCC=0.8677), (2) encoding providing the best specificity (AUC=0.9338, MCC=0.6474). These encodings are shown in Table 2 and Table 3, respectively.

In order to evaluate the efficiency of our algorithm, we calculated four performance measures after 5-fold cross-validation procedure for all amino acid encodings: specificity, sensitivity, Matthew’s Correlation Coefficient (ϕ coefficient) and Area Under the Curve (AUC). The measures were characterized by very small variance (see for example Table 1), which indicates the credibility of the applied cross-validation with 60 repetitions. All the encodings of amino acids showed very good and quite narrow range of AUC (0.93–0.97) and specificity (0.92–0.96). However, the sensitivity was characterized by much wider variation and ranged from 0.66 to 0.94 (Figure 3). For the final signalHsmm algorithm, we selected the encoding that yielded the highest sensitivity and the largest Matthew’s Correlation coefficient as well as the second best AUC (Table 2).

### 2.2. Comparison of Amino Acid Encodings

**Figure 4:**
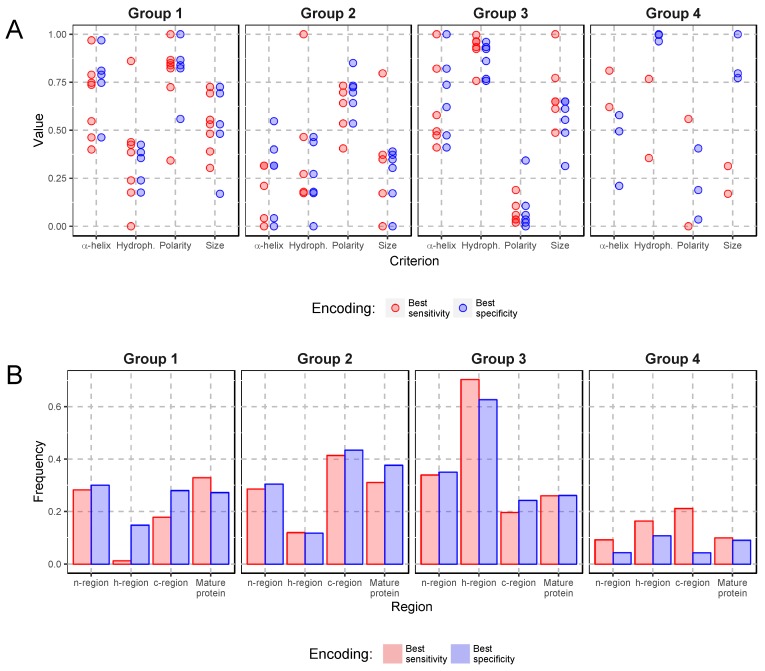
Comparison of amino acids classified into four groups providing the best sensitivity and specificity in the SP recognition. Normalized value of properties for particular amino acids represented by points (**A**). Frequencies of amino acids from the four groups in different regions of signal peptide and mature proteins (**B**).

We examined in detail the composition of encodings and properties of their amino acids with the best sensitivity and specificity (Table 2 and Table 3 and Figure 4). In both cases, group 1 tends to contain generally average-sized polar amino acids. This group is more uniform in the best sensitivity encoding because it includes all charged amino acids, both acidic and basic and also weakly basic histidine, whereas in the best specificity encoding, it does not have histidine, aspartic acid and its amide but contains alanine. These amino acids are nearly absent from the h-region and provide a very good distinction between the SP regions (Figure 4). In the best specificity encoding, for which the polar and charged character of group 1 is not so explicit, the difference in their distribution between the regions is also less visible.

Amino acids belonging to group 2 generally show quite a low probability of occurrence in α-helix. The best sensitivity encoding comprises two types of amino acids: all three hydroxylated residues as well as aliphatic glycine and proline known to break α-helices. The best specificity encoding lacks tyrosine but additionally includes aspartic acid and its amide, which increase the polar character of the group. Despite these differences, the occurrence of group 2 in both encodings is very similar in all SP regions (Figure 4). This group is the rarest in h-region and the most frequent in c-region.

Both encodings have strongly non-polar and aliphatic amino acids in group 3, such as: isoleucine, leucine, methionine and valine. The hydrophobic property of this group is pronounced in the best sensitivity encoding by the presence of aromatic tryptophan and phenylalanine, whereas the group 3 in the best specificity encoding includes also hydrophobic cysteine and slightly basic but aromatic histidine. Because of the hydrophobic character, this group dominates in the h-region in both amino acid classifications (Figure 4).

Group 4 is the most diverse in both encodings. In the case of the best sensitivity encoding, this group comprises only alanine and cysteine, which are rather small amino acids and tend to appear in α-helices. This very unique composition seems to be the most typical of the SP c-region. In contrast, group 4 in the best specificity encoding contains large aromatic amino acids: phenylalanine, tryptophan and tyrosine without special preference to the SP regions.

The encodings of amino acids play a crucial role in the recognition of SPs but do not affect to such an extent the identification of proteins without the SP. The change in the specificity for different encodings is seven times smaller than for sensitivity (Figure 3). It results from more uniform distribution of different residues in the mature protein than in the SP regions.

### 2.3. Benchmark Tests

**Figure 5:**
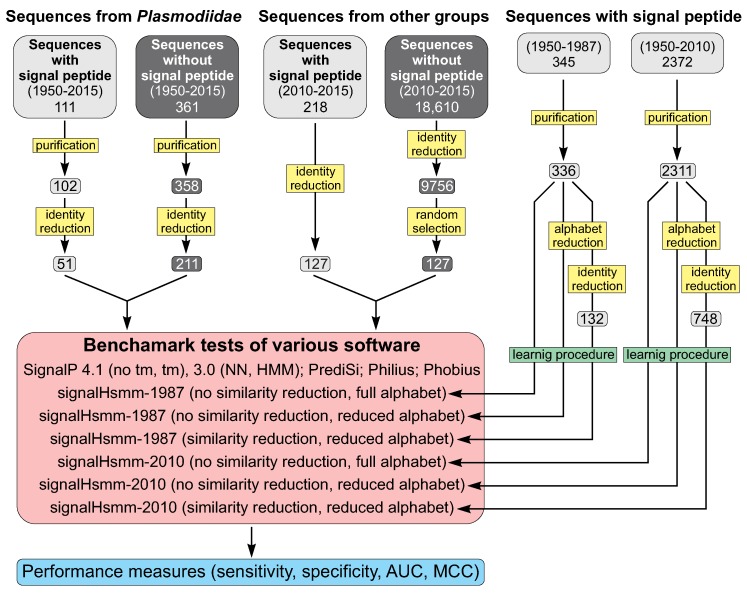
Data selection, training, testing and evaluation of signalHsmm.

In order to provide fair comparison of our algorithm with the previous software, we trained our model on 2311 SP-bearing sequences deposited in Uniprot until 2010 (the iteration of signalHsmm called signalHsmm-2010) (Figure 5). The set corresponded to the data used to train SignalP 4.1, the newest classifier present in the benchmark. In addition, we prepared a smaller dataset, covering only 336 sequences collected until 1987, which is the year the first method predicting SPs was published [45]. The signalHsmm-1987 iteration had to extract the SP model from the dataset more limited than the training sets used by all the classifiers included in the benchmark.

Together with signalHsmm, we evaluated several SP predicting algorithms in terms of recognition of atypical SPs from malaria parasites: SignalP 4.1, PrediSi, Phobius and Philius (see Table 4 for the most common performance measures, and Supplemental Table S1 for 23 performance measures). The older version of SignalP, i.e., SignalP 3.0, was also incorporated in the analysis because it is often chosen over SignalP 4.1 in the analysis of sequences belonging to the Apicomplexa due to its larger sensitivity [46,47]. In this comparison, our algorithm obtained the greatest AUC, MCC and maximal sensitivity. For 15 of 23 performance measures, signalHsmm was the best of all (Supplemental Table S1). PrediSi received the best specificity but at the expense of significantly reduced sensitivity.

We trained several iterations of signalHsmm described above to check improvements introduced to our software, i.e., the new probabilistic model (hidden semi-Markov model) and the simplified alphabet of amino acids. The latter was compared with the version trained on raw amino acid sequences, denoted as ‘raw aa’ in Table 4. The performance of this iteration was mostly worse than the performance of its counterpart trained on the reduced alphabet. The version with the amino acid encodings outperformed the version with simple amino acids in 17 of 23 measures (Supplemental Table S1). The obtained results confirm that the function of the SP does not depend on specific amino acids but on more general features, and our simpler model is able to recognize the unique structure of the SP more accurately. The advantage of the hidden semi-Markov model over normal Markov model was presented through the comparison of signalHsmm with SP predictors utilizing HMM: Phobius, Philius and SignalP 3.0 (HMM)—Table 4. For 17 of 23 measures including AUC, MCC and sensitivity, our algorithm outperformed all of them (Table 4, Supplemental Table S1).

In order to check the susceptibility of our model to overfitting, we trained it on a dataset with 50%-sequence identity reduction. We discovered that our model, probably thanks to its relative simplicity, did not overfit and versions trained on the set with and without the restrictive sequence identity reduction were comparable. Moreover, the former was slightly better in 21 measures than the model based on all sequences.

The overall simplicity of our approach does not hinder its capabilities to recognise SPs from other organisms. We benchmarked signalHsmm iterations and other software on the set of 127 eukaryotic proteins with and without SPs that were added to the UniProt database after 2010. Their sequence identity in the testing set was also reduced as described above. SignalHsmm-2010 performed comparably to SignalP (see Supplemental Table S2 for all performance measures). Its AUC was 0.94 compared to 0.95 and 0.96 of the two SignalP versions. For 20 measures, it was the second in the ranking just after SignalP programs and, for five parameters (sensitivity, recall, true positive rate, the number of true positives and false negatives), it outperformed the newest version of SignalP.

### 2.4. Specific Composition of *Plasmodiidae* Signal Peptides

**Figure 6:**
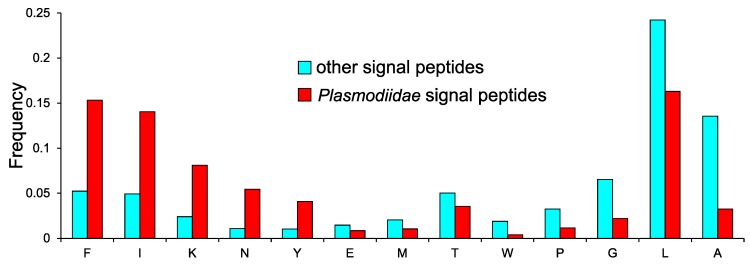
Mean frequency of amino acids that significantly discriminate SPs from *Plasmodiidae* and other organisms. Amino acids were arranged according to the difference in the mean values.

The plasmodial genomes are characterized by a large excess of adenine and thymine [18], and this strongly influences the amino acid composition of their encoded proteins including SPs (Figure 6). SPs differ significantly between *Plasmodiidae* and other taxa in composition of 13 amino acids (Wilcoxon test, *p* < 0.05 corrected by the Benjamini–Hochberg method in the multiple testing). The plasmodial SPs are especially abundant in amino acids coded by codons rich in adenine and thymine, such as: phenylalanine (TTY), isoleucine (ATH), lysine (AAR) and asparagine (AAY), whereas they are poor in amino acids coded by guanine and cytosine rich codons: alanine (GCN), glycine (GGN) and proline (CCN). Leucine is coded by a set of codons with mixed composition (TTR, CTN) but also discriminates the two sets of SPs.

**Figure 7:**
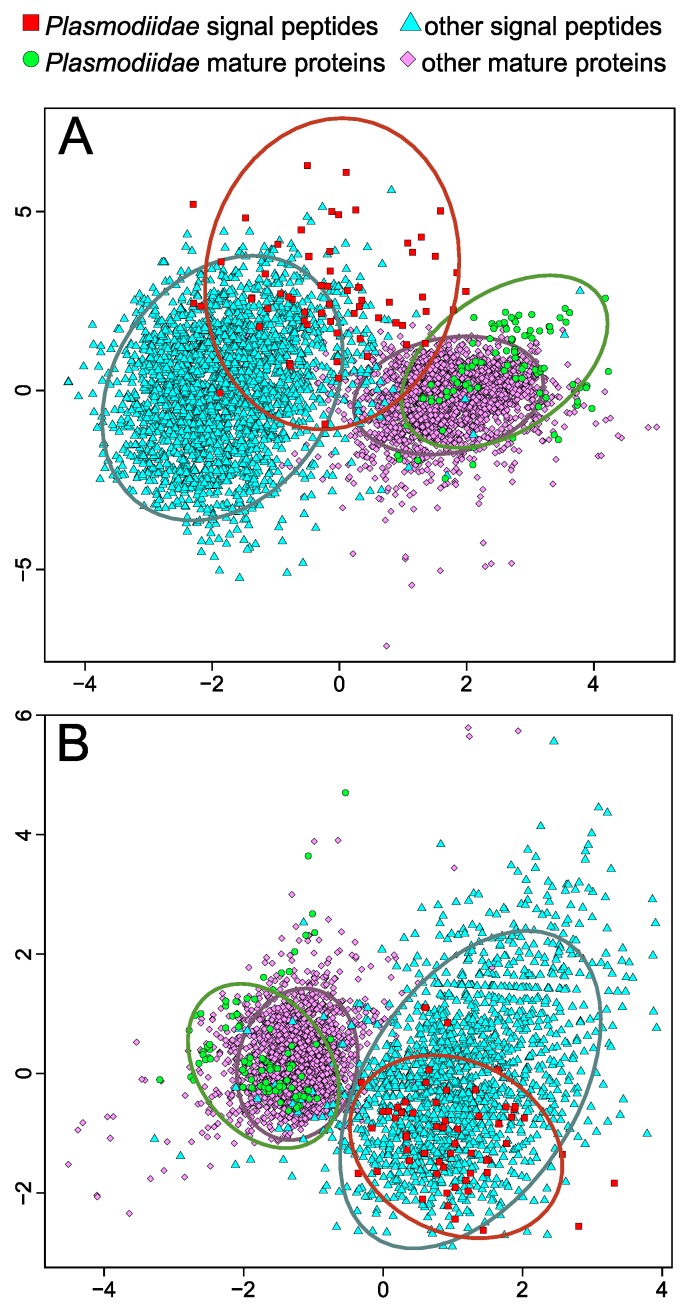
Principal Component Analysis performed on raw amino acid composition (**A**) and amino acids encoding into groups (**B**) for SPs and mature proteins from *Plasmodiidae* and other organisms. SPs from *Plasmodiidae* create a set, which is quite distinct from other SPs according to raw amino acid composition. However, the application of amino acid encoding chosen in cross-validation makes these sets similar but still supporting a significant difference between SPs and mature proteins. Concentration ellipses cover 95% of points from a given set.

As a result, plasmodial SPs separate from other SPs according to raw amino acid composition in Principal Component Analysis (Figure 7A). Mature proteins of *Plasmodiidae* are also shifted in the plot from other mature proteins. Therefore, algorithms that consider only particular amino acids may not have decision rules appropriate for such composition and consequently fail to identify SPs. Interestingly, the amino acid encoding employed by signalHsmm reduces this difference and unifies all SPs into one set (Figure 7B). Similarly, when the reduced amino acid alphabet is considered, mature proteins are also inseparable in the plot. It should be emphasized that the reduction of the amino acid alphabet used by our algorithm does not weaken the difference between SPs and mature proteins, which still create distinguishable groups. These analyses indicate that the applied amino acid encoding enables capturing a general composition of many SPs keeping simultaneously their difference from mature proteins. The use of physicochemical properties of amino acids instead of raw sequences also improved the prediction of protein function in four representative model organisms: *Homo sapiens*, *Arabidopsis thaliana*, *Saccharomyces cerevisiae* and *Mycobacterium tuberculosis* [48].

## 3. Materials and Methods

### 3.1. Overview

Since the functionality of SPs depends on the physicochemical properties of residues in a given SP region, we clustered amino acids into several groups based on this criterion. The pre-processed sequences were further analysed by an enhanced version of a heuristic algorithm employed in SignalP 2.0, which determines borders between the three SP regions [41]. We also refined some criteria for recognition of the regions to attune the algorithm to atypical SPs. Next, two models were trained to detect proteins with and without the SP. The first one was a hidden semi-Markov model, in which each of the three SP regions was represented by a different hidden state. The additional fourth hidden state represented the mature protein. Each state was described by its frequencies of amino acid groups. The distribution of hidden states durations, i.e., the number of amino acids, was based on the empirical distribution of region lengths from the training set. Furthermore, the hidden semi-Markov model was enriched with n-grams representing SP cleavage sites. The second model was a simple probabilistic approach in which no association between amino acids was assumed and the probability of presence of amino acids groups was determined by their frequencies in mature proteins.

### 3.2. Data Selection

The final predictor was trained on 2438 experimentally confirmed SPs from eukaryotic proteins, which sequences and annotations were downloaded from the UniProt database release 2015_06. We removed sequences with more than one cleavage site, an unknown cleavage site and ambiguous symbols of amino acid residues: X, J, Z, B and U (selenocysteine). Sequences without the SP were randomly selected in the same number as the positive set. Moreover, we created a learning subset of 2311 sequences deposited in the database until 2010 to perform fair comparison of our algorithm with older software that was trained on smaller number of sequences (Figure 5). We also used a subset of 336 sequences present in the database until 1987, which is the year the first method predicting the SP was published, to check susceptibility of our algorithm to a limited amount of information.

The main testing set consisted of proteins from the *Plasmodiidae* family (Figure 5). The positive set contained 102 sequences with a putative SP with the annotated start and cleavage site. The corresponding negative set comprised 358 sequences without any SP information. The other testing set consisted of 127 eukaryotic proteins with an SP included in the UniProt database after 2010.

### 3.3. Sequence Identity Reduction in Studied Sequence Sets

In order to reduce the set according to identity of collected protein sequences, we filtered them using cd-hit [49]. SP sequences and the first 70 amino acid residues of proteins without the peptide were subjected to sequence identity reduction according to Nielsen et al. [50]. We prepared two reduced learning datasets with sequences deposited until 2010 and 1987 by removing sequences with identity larger than 50% threshold (word length 2). After this procedure, the sets contained 748 and 132 sequences with SPs, respectively. The main *Plasmodiidae* set was filtered in the same manner and reduced to 51 and 211 sequences with and without the SP, respectively.

### 3.4. Clustering of Amino Acids into Groups

In order to reduce the alphabet of amino acids, we clustered them into several physicochemical groups, essentially re-using our methodology from a previous study [51]. It is a different approach compared to BLOMAP [42], which also uses a reduced alphabet of amino acids, but based on substitution matrices. We grouped amino acids using four properties relevant for the structure of the SP: their hydrophobicity, tendency to build α-helices, polarity and size. The high hydrophobicity is a good determinant of the h-region, which α-helix secondary structure is probably induced by the positively charged n-region. The high polarity as well as small size are important features of residues in the c-region and cleavage site [11].

We considered in total 13 amino acid scales present in AAIndex database [65] (Table 5). We selected one scale per a given property and carried out all possible 96 permutations of them. Based on that, we created 96 possible clusterings of amino acids using Euclidean distance and Ward’s method. Next, we cut the clusterings to create four groups of amino acids. In 31% of cases, the groupings were identical. To compare the usefulness of these encodings, we performed a 5-fold cross-validation training of our algorithm on every encoding. We created balanced data sets by subsampling proteins without the SP to equal the number of proteins with the SP. The cross-validation was repeated 60 times to ensure that every protein without the SP was included in the learning set with the probability higher than 0.5.

### 3.5. Hidden Semi-Markov Model

Our algorithm is based on a hidden semi-Markov model (HSMM), which is an extension of the hidden Markov model (HMM) [66,67,68]. The HMM consists of two stochastic processes. The first is a discrete Markov chain $X_{t=1}^{T}$ on the set of hidden states $\left\{S_{1},\ldots S_{n}\right\}$, where *t* means a step of this process and *T* means total duration of the process corresponding to the length of the SP. Hidden states represent particular SP regions and are ‘the cause’ of the observations, which are amino acid residues in analysed sequences. In a subsequent step t+1, the hidden state might change to another according to a transition matrix $A={\left(a\right)}_{i,j=1}^{n}$, where $a_{i,j}=P\left(X_{t+1}=S_{j}|X_{t}=S_{i}\right)$ means a probability of being in a state *j* on condition that in the previous step was a state *i*. The second process $E_{t=1}^{T}$ is an observation process defined on the set of possible observations $\left\{O_{1},\ldots ,O_{m}\right\}$. They are assumed to occur independently but conditionally on the hidden states that emits these observations. Distribution of the observations are given by a matrix $B={\left(b\right)}_{i,k=1}^{m}$, where $b_{i,k}=P\left(E_{t}=O_{k}|X_{t}=S_{i}\right)$ means a probability of emission of observation *k* on condition that a hidden state was *i*. The main goal of signalHsmm is to find the most probable SP regions boundaries for a given sequence. This is achieved with the Viterbi algorithm.

In the regular HMM, the hidden state duration, i.e., the number of observations emitted by the hidden state, has a geometric distribution. Durbin et al. [69] showed how to extend it for different distributions without significant increase in computational complexity. Similar ideas were used for SP recognition, for example by Käll et al. [38]. However, it is still not flexible enough because the empirical regional length distributions (see Figure 2) are difficult to capture in this way.

**Figure 8:**
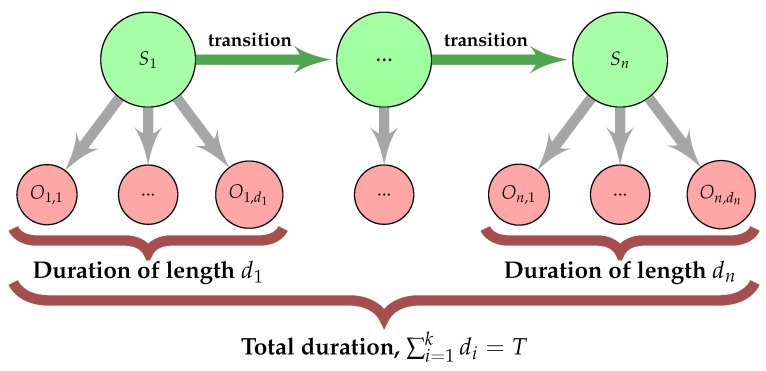
General scheme of hidden semi-Markov model. The model consists of hidden states *S* representing regions of the SP with the length distribution given by the state duration *d*. The hidden states emit observations *O* which are amino acid residues.

In our approach, we used a modification of HMM called hidden semi-Markov model (HSMM) [67]. It extends the HMM by assuming a duration distribution for a given hidden state (Figure 8). Then, the model includes additionally probabilities of duration in hidden states:

$$P(\mathrm{duration}\;\mathrm{in}\;\mathrm{state}=d|\mathrm{state}\;\mathrm{is}\;S_{i}),\;\;i=1,\ldots ,n,\;\;d=1,\ldots ,D,$$

where *D* is the maximum allowed duration. Since our data sets are sufficiently large and *D* is small—around 30 amino acid residues, computational effort is not much higher than in the regular HMM.

**Figure 9:**
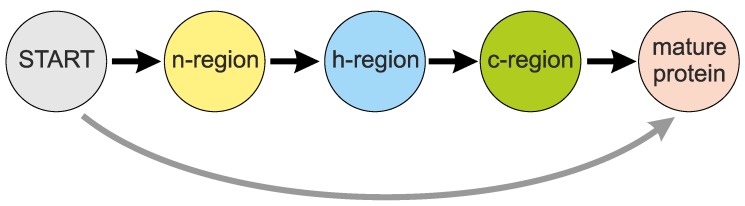
Diagram of signalHsmm showing hidden states which represent the SP regions and mature protein. Arrows indicate transitions between these states.

Almost all entries in the transition matrix *A* are zeros because regions represented by hidden states are sequential. The possible transitions between them are depicted as arrows in Figure 9. The probabilities of observations for the hidden states and hidden states durations were estimated from the training data. The advantage of the HSMM model results not only from its better performance but also form its straightforwardness and flexibility.

## 4. Conclusions

We proposed a novel solution to the problem of SP prediction, which is very efficient in recognition of atypical SPs from plasmodial proteins despite the fact that the program was trained on data coming from all eukaryotes. It indicates that our algorithm is able to describe common features of all SPs based on the classical division of the SP into three regions. Our software is not limited to very specific taxonomic group, and is able to compete with state-of-art algorithms in detecting SPs of other organisms.

One of the most important features of signalHsmm is its stability. The difference in performance for versions trained on large and small datasets deposited in databases at different times is negligible. It implies that signalHsmm, thanks to its unique structure, extracts roughly the same general information from SPs regardless of the size and type of the training dataset. Similarly, iterations trained on datasets with and without the removal of redundancy, resulting from sequences homology, showed similar prediction efficiency. For the first dataset, the algorithm was even slightly better. It suggests that our probabilistic model is quite resistant to overfitting and does not adjust itself to the most common patterns in the training dataset but retrieves the universal SP model.

The existing software detecting SPs does not usually reveal decision rules responsible for the prediction. Our algorithm is the first step to explicitly show features of SPs important in their recognition, which is interesting from the biological point of view. The applied encoding of amino acids not only reduces the dimensionality of the problem, but also makes our probabilistic model more interpretable. Thanks to that, we were able to determine physicochemical properties of amino acids for particular SP regions. Our model confirmed not only the high hydrophobicity of the h-region and polarity of the n-region but also found that hydroxylated amino acids are one of the most typical amino acids in the c-region. In contrast to the h-region, it also contains α-helix breakers: glycine and proline.

The flexibility and efficiency in recovering information makes signalHsmm unique among similar software. It properly models very specific SPs belonging to narrow taxonomic groups that are poorly represented in databases and can effectively extract information from very small datasets. Our approach may lead in future to development of new predictors specialized in recognition of atypical signals targeting sequences to variuos subcellular compartments.

The prediction of SPs is of great importantance as proteins equipped with these targeting signals are involved in many processes associated with diseases and disorders. Therefore, better knowledge and prediction of SPs can enable scientists to discover new drugs and development of more efficient therapies. Particularly, good drug targets could be proteins containing SPs that are targeted to plasmodial specific compartments such as apicoplasts.

## Figures and Tables

**Table 1:** Performance measures for the amino acid encoding with the highest sensitivity calculated for 60 repetitions of cross-validation.

Measure	Mean	SD
AUC	0.9682	0.0023
Sensitivity	0.9407	0.0008
Specificity	0.9272	0.0050
MCC	0.8681	0.0049

**Table 2:** The encoding of amino acids with the best sensitivity.

Group	Amino Acids
1	D, E, H, K, N, Q, R
2	G, P, S, T, Y
3	F, I, L, M, V, W
4	A, C

**Table 3:** The encoding of amino acids with the best specificity.

Group	Amino Acids
1	A, E, K, Q, R
2	D, G, N, P, S, T
3	C, H, I, L, M, V
4	F, W, Y

**Table 4:** Comparison of Sensitivity, Specificity, Matthews Correlation Coefficient (MCC) and Area Under the Curve (AUC) for different classifiers and signal peptide-bearing proteins from members of *Plasmodiidae*. The abbreviations ’tm’ and ’no tm’ indicate version considering and not considering transmembrane domains, whereas ’ident. 50%’ means removal from the learning set sequences with the sequence identity larger than 50%.

	Sensitivity	Specificity	MCC	AUC
SignalP 4.1 (no tm) [33]	0.8235	0.9100	0.6872	0.8667
SignalP 4.1 (tm) [33]	0.6471	0.9431	0.6196	0.7951
SignalP 3.0 (NN) [40]	0.8824	0.9052	0.7220	0.8938
SignalP 3.0 (HMM) [40]	0.6275	0.9194	0.5553	0.7734
PrediSi [37]	0.3333	**0.9573**	0.3849	0.6453
Philius [39]	0.6078	0.9336	0.5684	0.7707
Phobius [38]	0.6471	0.9289	0.5895	0.7880
signalHsmm-2010	0.9804	0.8720	0.7409	0.9262
signalHsmm-2010 (ident. 50%)	**1.0000**	0.8768	**0.7621**	**0.9384**
signalHsmm-2010 (raw aa)	0.8431	0.9005	0.6853	0.8718
signalHsmm-1987	0.9216	0.8910	0.7271	0.9063
signalHsmm-1987 (ident. 50%)	0.9412	0.8768	0.7194	0.9090
signalHsmm-1987 (raw aa)	0.7647	0.9052	0.6350	0.8350

**Table 5:** Properties of amino acids used in their clusterization.

Property Name	Amino Acid Scale
Size	Size [52]
Size	Molecular weight [53]
Size	Residue volume [54]
Size	Bulkiness [55]
Hydrophobicity	Normalized hydrophobicity scales for α-proteins [56]
Hydrophobicity	Consensus normalized hydrophobicity scale [57]
Hydrophobicity	Hydropathy index [58]
Hydrophobicity	Surrounding hydrophobicity in α-helix [59]
Polarity	Polarity [60]
Polarity	Mean polarity [61]
Occurrence in α-helices	Signal sequence helical potential [62]
Occurrence in α-helices	Normalized frequency of N-terminal helix [63]
Occurrence in α-helices	Relative frequency in α-helix [64]

## Supplementary Materials

Supplementary materials can be found at http://www.mdpi.com/1422-0067/19/12/3709/s1. Table S1: **Benchmark results—*Plasmodiidae***. Performance measures for benchmark test of SP predictors using sequences belonging to *Plasmodiidae*. Table S2: **Benchmark results—Eukaryotes**. Performance measures for benchmark test of SP predictors using all eukaryotic sequences. The signalHsmm prediction web-server is available at: http://smorfland.uni.wroc.pl/shiny/signalHsmm/. SignalHsmm is implemented as an R package available at: https://cran.r-project.org/package=signalHsmm. The stand-alone version offers prediction and tools to build, train and test novel SP models.

## Abbreviations

The following abbreviations are used in this manuscript:

SP	Signal Peptide
HSMM	Hidden Semi-Markov Model
HMM	Hidden Markov Model
AUC	Area Under the Curve
MCC	Matthews Correlation Coefficient
