# Supplementary material for: Prediction of Signal Peptides in Proteins from Malaria Parasites
Source: Int J Mol Sci. 2018 Nov 22;19(12):3709. doi: 10.3390/ijms19123709 (PMC6321056; doi:10.3390/ijms19123709)
Supplement: Supplementary file 1 [file ijms-19-03709-s001.zip › ijms-385348 suppl/supplementary.docx]

**Availability and Implementation**

The signalHsmm prediction web-server is available at: http://smorfland.uni.wroc.pl/shiny/signalHsmm/.

signalHsmm is implemented as an R package available at:

https://cran.r-project.org/package=signalHsmm.

The stand-alone version offers prediction and tools to build, train and test novel SP models.
